# Impact of gut microbiome on radiotherapy and immunotherapy efficacy in microsatellite-stable colorectal cancer: role of propionic acid and *B. fragilis*

**DOI:** 10.1038/s41416-025-03105-2

**Published:** 2025-07-26

**Authors:** Lu Yu, Qiqing Guo, Xinyi Gu, Zihuan Wang, Jiaying Li, Xusheng Wang, Zi Xu, Yafang Wang, Yuqin Zhang, Yaowei Zhang, Yanqing Ding, Zhenhui Chen, Keli Chen, Yi Ding

**Affiliations:** 1 Department of Radiation Oncology, Nanfang Hospital, Southern Medical University, Guangzhou, China; 2 Department of Ultrasound, Shanghai Ninth People’s Hospital, Shanghai Jiaotong University School of Medicine, Shanghai, China; 3 Department of Gastroenterology, Xinqiao Hospital, Army Medical University, Chongqing, China; 4 Department of Radiation Oncology, National Cancer Center/National Clinical Cancer Research Center for Cancer/Cancer Hospital & Shenzhen Hospital, Chinese Academy of Medical Sciences and Peking Union Medical College, Shenzhen, China; 5 Department of Radiotherapy, General Hospital of Southern Theatre Command, Guangzhou, China; 6 Guangdong Province Key Laboratory of Molecular Tumor Pathology, Guangzhou, China; 7 Department of Microbiology, Guangdong Provincial Key Laboratory of Tropical Disease Research, School of Public Health, Southern Medical University, Guangzhou, China; 8 HuiQiao Medical Center, Nanfang Hospital, Southern Medical University, Guangzhou, China

**Keywords:** Colorectal cancer, Cancer metabolism, Cellular microbiology

## Abstract

**Background:**

Colorectal cancer (CRC) is the third most common cancer and the second leading cause of cancer-related deaths worldwide. While immunotherapy is effective in microsatellite instability-high (MSI-H) CRC, its benefits in microsatellite-stable (MSS) CRC are limited. Radiotherapy may modify the immune microenvironment in MSS-CRC, enhancing immunotherapy efficacy, but individual responses vary.

**Methods:**

We employed MSS-CRC mouse models to examine the effects of combined radiotherapy and immunotherapy, with and without antibiotics (ABX). Various analyses, including metagenomic, nontargeted metabolomic, and gas chromatography-mass spectrometry (GC-MS), were performed to identify factors influencing treatment outcomes. Flow cytometry, immunohistochemistry and in vivo antibody blockade experiments assessed the role of metabolites and bacteria on CD8^+^ T cell infiltration and treatment responses, complemented by transcriptomic sequencing and molecular biology experiments.

**Results:**

Our analyses identified propionic acid and *Bacteroides fragilis* (*B. fragilis*) as crucial factors enhancing the efficacy of combined therapies in MSS-CRC. Both propionic acid and *B. fragilis* improved CD8^+^ T cell infiltration and treatment outcomes, with molecular assays indicating that propionic acid facilitates H3K14 acetylation, activating the Meox1-Cxcr6/Ccl5 axis.

**Conclusions:**

This study highlights the pivotal role of the gut microbiome, specifically propionic acid and *B. fragilis*, in modulating the efficacy of combined radiotherapy and immunotherapy in MSS-CRC.

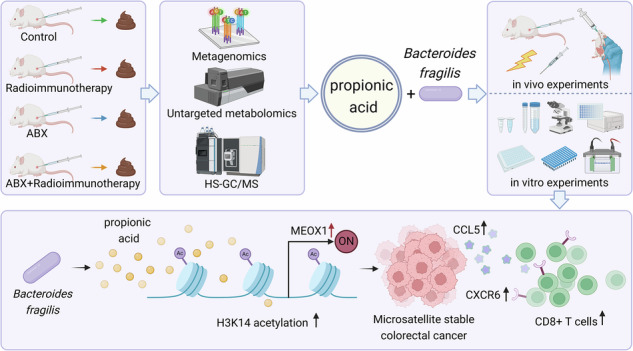

## Introduction

Cancer immunotherapy has been notably transformed by the introduction of immune checkpoint inhibitors, which have been effective in microsatellite instability (MSI)-high colorectal cancer (CRC) [1–3]. In these cases, immunotherapy can induce a near-complete response, offering significant therapeutic benefits [4–6]. However, the efficacy and response rates to immunotherapy in microsatellite-stable (MSS) CRC remain low [1, 7], thus warranting the need for innovative strategies to enhance the efficacy of immunotherapy in MSS-CRC.

The potential of combining immunotherapy with radiotherapy to overcome the limitations of single-agent immunotherapy has been highlighted [8]. Radiotherapy can expose local tumour antigens and improve the tumour microenvironment [9, 10], facilitating the infiltration of immune cells, particularly effector CD8^+^ T cells, into the tumour site [11, 12]. Combining radiotherapy with immunotherapy increases the visibility of tumour antigens and enhances the overall immune response against the tumour [12, 13]. This combinatorial approach has been supported by recent clinical trials evaluating neoadjuvant chemoradiotherapy or radiotherapy combined with immunotherapy [14, 15]. These studies have demonstrated encouraging outcomes, including improved complete response rates, suggesting the valuable role of combinatorial strategies in enhancing therapeutic efficacy.

Additionally, the gut microbiota has been found to influence cancer progression and therapeutic responses [16, 17]. The gut microbiome, a complex and dynamic ecosystem of microorganisms in the gastrointestinal tract, has been associated with tumour biology and treatment outcomes [18, 19]. Emerging evidence indicates that the gut microbiota can directly or indirectly affect tumour development and response to therapy [20, 21]. Notably, the microbiome significantly affects the efficacy and safety of cancer immunotherapy [17, 22]. Variations in microbial composition and functionality modulate immune responses and potentially influence the success of immunotherapy [22].

The production of microbial metabolites is a crucial mechanism underlying the interaction between the gut microbiota and the host [23]. Short-chain fatty acids (SCFAs) are prominent metabolites produced by the gut microbiota through the fermentation of dietary fibres [24]. SCFAs, including acetate, propionate, and butyrate, regulate host immune responses and maintain gut health [24, 25]. Specifically, propionate, produced by specific gut bacteria such as *Bacteroides* and *Clostridium* [24, 26], influences various physiological processes, including immune regulation and inflammation [27, 28].

Propionate acts through several mechanisms, including the modulation of histone acetylation [29, 30], by promoting the activity of acetyltransferases or inhibiting histone deacetylases (HDACs) [31, 32]. This epigenetic modification can influence gene expression and contribute to changes in immune responses and tumour microenvironment [30, 32]. By altering histone acetylation, propionate and other SCFAs may enhance the efficacy of cancer treatments, including immunotherapy and radiotherapy [17, 29, 33]. The ability of propionate to modulate histone acetylation and subsequent gene transcription highlights its potential as a therapeutic adjunct in cancer treatment.

The interaction between the gut microbiota, microbial metabolites, and neoadjuvant radiotherapy and immunotherapy has not been well understood, requiring further investigation. Understanding the mechanism by which the gut microbiota and microbial metabolites influence immune responses and tumour biology can provide valuable insights into optimising cancer treatments.

This study aims to elucidate how gut microbiota and their metabolites influence neoadjuvant radiotherapy-immunotherapy responses in MSS-CRC and identify specific microbial and metabolic enhancers of this combined treatment. Through the systematic investigation of microbiome-metabolite-immune crosstalk, we used metagenomic sequencing and gas chromatography-mass spectrometry (GC-MS) metabolomics to identify propionic acid and *Bacteroides fragilis* (*B. fragilis*) as crucial modulators of treatment efficacy in antibiotic-treated mice versus untreated mice. Functional validation through in vivo antibody blockade, flow cytometry, and immunohistochemistry (IHC) confirmed their roles in enhancing CD8^+^ T cell infiltration and therapeutic outcomes. Mechanistically, we demonstrated that propionic acid induces H3K14 acetylation to activate the Meox1-Cxcr6/Ccl5 axis via integrated transcriptomic and molecular biology experiments. These findings reveal actionable microbial-metabolic targets and provide novel strategic insights for enhancing combined radiotherapy-immunotherapy efficacy in MSS-CRC.

## Materials and methods

### Cell culture and bacterial culture

CT26 cell line was provided by the Department of Pathology of Nanfang Hospital with STR profiling and cultured in Dulbecco’s Modified Eagle’s Medium (DMEM, Gibco, Grand Island, NY, USA) supplemented with 10% foetal bovine serum (FBS, Gibco) at 37 °C under 5% CO_2_. *B. fragilis* was purchased from the ATCC (# 25285) and cultured in basal liquid brain heart infusion (BHI) medium (Huaikai Bio-Technology, Guangzhou, China) at 37 °C for 48 h under a strict anaerobic environment (90% N_2_ and 10% H_2_). In the co-incubation experiment with CT26 cells, CT26 cells were seeded into 6-well plates (Nest, Wuxi, China) and cultured until a density of 90% was achieved. The IRT group was established by exposing the cells to 6 Gy of irradiation and incubating them with 40 µg of InVivo MAb anti-mouse PD-1 (CD279, B10421D1, BioXcell, Lebanon, NH, USA). In the PR + IRT group, 100 mmol of sodium propionate (P5436, Sigma–Aldrich, St. Louis, MO, USA) was added to the culture medium and co-incubated for 24 h before the cells were harvested. In the FRA + IRT group, 1 × 10^7^ CFU of *B. fragilis* was co-incubated with the cells for 4 h and washed with phosphate-buffered saline (PBS) thrice. After 20 h of culture, the cells were collected for analysis.

### Animal model

All animal experiments were approved by the Institutional Animal Care and Use Committee of the Nanfang Hospital, Southern Medical University. Four- to six-week-old male BALB/c mice were purchased from Zhiyuan Biotechnology Company (Guangzhou, China) and raised under specific pathogen-free conditions with free access to food and drinking water. All in vivo experiments were performed in accordance with our institution’s guidelines. The mice were allowed to adapt to the experimental environment for one week and randomly categorised without blinding. Animal sample sizes were determined via power analysis to ensure rigour, reproducibility, and ethical animal use.

To develop the subcutaneous tumour model, 3 × 10^6^ cells were subcutaneously injected into the right flank of the mice. At a tumour volume of 50 mm^3^, different treatments were performed. For radiation (RT) treatment or radiotherapy-immunotherapy (IRT), the tumours were irradiated once at 6 Gy (Varian Clinac 23EX Linear Accelerator, USA), and the other parts of the mouse’s body were protected with a lead shield. For the immunotherapy (ICI) or IRT, mice were injected once with 100 μg of anti-mouse PD-1 antibody (CD279, B10421D1, BioXcell) intraperitoneally. For the ABX treatment, mice were orally administered an ABX cocktail of ampicillin (1 g/L, A8180, Macklin, Shanghai, China), streptomycin (1 g/L, AS325, Sigma–Aldrich), metronidazole (1 g/L, M8060, Macklin), and vancomycin (0.25 g/L, V2002, Sigma–Aldrich) daily when the cells injected. For the CD8 antibody (CD8i) treatment or IgG control antibody (IgG), mice were injected ‌intraperitoneally with 200 μg of InVivoMab anti-mouse CD8α (A2102, Selleck, Houston, TX, USA) or Rat IgG2b isotype control-InVivo (A2116, Selleck) every four days. For the propionic acid (PR) treatment, mice were orally administered 200 mmol/L of sodium propionate (P5436, Sigma–Aldrich) in 200 μL of PBS. For the *B. fragilis* (FRA) treatment, mice were orally administered 1 × 10^9^ CFU of *B. fragilis* in 200 μL of PBS. For the in vivo Meox1-siRNA treatment, mice were intratumorally injected with 1 nmol/20 g of Meox1-siRNA every 3 days. The in vivo Meox1-siRNA (5’-GGAGGATTGCATGGTACTTGG-3’) and Control-siRNA were purchased from Guangzhou RiboBio Co., Ltd. (Guangzhou, China). Tumour volume was measured using vernier callipers and calculated as 1/2 × length × width × width [34]. After the mice were humanely euthanised with phenobarbital sodium, with death confirmed by absence of vital signs, and the tumours were excised and stored for further experiments.

### Flow cytometry

Flow cytometry was conducted as previously reported [35]. Subcutaneous tumours were collected and washed once with pre-chilled HBSS buffer (Gibco). Tumours were homogenised on ice with ophthalmic scissors in 200 µL of DMEM (Gibco). Subsequently, 1 mL of DMEM containing 0.5 mg/mL collagenase IV (C8160, Solarbio, Beijing, China) and 0.1 mg/mL DNase I (D8071, Solarbio) was added, and the mixture was incubated at 37 °C with shaking for 30 min until a soup-like consistency was achieved. Digestion was halted by adding three times the volume of DMEM containing 10% foetal bovine serum (Gibco). The mixture was filtered through a 70 µm cell strainer (Biologix, Wuhan, China) and centrifuged at 4 °C, 350 × *g* for 5 min to obtain a cell pellet. The pellet was resuspended in 1 mL of ACK lysis buffer (CS0001, Leagene, Beijing, China), gently mixed, and incubated at room temperature for 1 min to lyse the red blood cells. The reaction was terminated by adding 3 mL of HBSS buffer. The mixture was then centrifuged at 4 °C, 350 × *g* for 5 min to obtain a cell pellet, which was washed twice with HBSS buffer. The cells were resuspended in 100 µL of HBSS and stained with Ms. CD45 APC-Cy7 (561037, BD Pharmingen, San Diego, CA, USA), FITC anti-mouse CD3 (100203, Biolegend, San Diego, CA, USA), Brilliant Violet 421 anti-mouse CD4 (100437, Biolegend), PE anti-mouse CD8a (100707, Biolegend), and APC anti-mouse CD49b (103515, Biolegend). Staining was performed in the dark for 30 min, followed by centrifugation of the cell pellet. The cells were then washed twice with HBSS. Finally, the cells were resuspended in 200 µL of liquid and filtered through a 40-µm cell strainer (Biologix) before analysis. Flow cytometry was conducted using BD Fortessa X-20 and FlowJo v10.6.2 for gating and data analysis.

### IHC staining

IHC staining of paraffin-embedded mouse tumour sections was performed as previously reported [34]. Sections were deparaffinized, rehydrated, subjected to antigen retrieval, and blocked with 3% hydrogen peroxide and goat serum, followed by overnight incubation with primary rabbit anti-GZMB antibody (ab255598, Abcam, Cambridge, UK, 1:3000) at 4 °C. The next day, the sections were incubated at room temperature for 30 min to rewarm, followed by incubation with the secondary antibody for 1 h at room temperature, and DAB staining was performed using an IHC assay kit (Maixin, China). Counterstaining was performed using hematoxylin for 2 min. Images were captured using an Olympus microscope (Evident Corporation, Tokyo, Japan).

### Metagenomics

Metagenomic analysis was conducted at Majorbio (Shanghai, China) following the manufacturer’s protocol [36]. The genomic DNA of the intestinal contents was extracted, assessed by agarose gel electrophoresis, and fragmented to ~350 bp using Covaris M220. Library construction involved adaptor ligation, removal of self-ligated fragments, PCR enrichment, and denaturation to produce single-stranded DNA. Bridge PCR was used to amplify the DNA clusters, which were linearised for sequencing. DNA was sequenced using modified DNA polymerase and fluorescent dNTPs, and nucleotide incorporation was recorded per cycle. They were sequenced using the Illumina NovaSeq 6000 platform to obtain high-quality sequencing data. Bioinformatics analysis processed raw data, assembled sequences, predicted genes, and annotated them using the NR and Kyoto Encyclopedia of Genes and Genomes (KEGG) databases on the Majorbio Cloud Platform (https://cloud.majorbio.com/) [37].

### Nontargeted metabolomics

Nontargeted metabolomic analysis was conducted at Majorbio (Shanghai, China) following the manufacturer’s protocol [36]. Approximately 0.1 g of intestinal content was collected, and metabolites were extracted with 80% methanol. All chromatographic separations were performed using an UltiMate 3000 UPLC System (Thermo Fisher Scientific, Waltham, MA, USA). An ACQUITY UPLC HSS T3 column (100 × 2.1 mm, 1.8 μm, Waters, Milford, MA, USA) was used for the reversed-phase separation. The column oven was maintained at 40 °C. Gradient elution conditions were set as follows: 0–0.8 min, 2% B; 0.8–2.8 min, 2–70% B; 2.8–5.6 min, 70–90% B; 5.6–6.4 min, 90–100% B; 6.4–8.0 min, 100% B; 8.0–8.1 min, 100–2% B; and 8.1–10 min, 2% B. A high-resolution tandem mass spectrometer (TripleTOF 6600; SCIEX, Framingham, MA, USA) was used to detect the metabolites eluted from the column. The Q-TOF instrument was operated in positive and negative ion modes. The acquired MS data were pretreated using XCMS software. The online KEGG database annotated the metabolites by matching the exact molecular mass data (m/z) of samples with those from the database.

### GC-MS

Standard solutions of acetic acid (71251, Sigma–Aldrich), propionic acid (94425, Sigma–Aldrich), butyric acid (19215, Sigma–Aldrich), isobutyric acid (46935u, Sigma–Aldrich), valeric acid (75054, Sigma–Aldrich), isovaleric acid (78651, Sigma–Aldrich), and caproic acid (21529, Sigma–Aldrich) were prepared at a concentration of 10 mmol/L each. The sample pre-treatment for GC-MS was improved based on a previous study [38]. Approximately 0.1 g of intestinal content was collected and homogenised with 500 μL of Millipore water and 3 mm ceramic beads (Beyotime, Hangzhou, China) on ice for 2 min, followed by 5 min of ultrasonication. The process was conducted using a sealing membrane to minimise the loss of SCFAs. The homogenate was transferred to a headspace vial (Agilent, Santa Clara, CA, USA), and the previous tube was rinsed with 500 μL of Millipore water and added to the vial. The vial was sealed and placed in a headspace shaker, which was heated to 80 °C for 30 min with shaking. A 1-mL sample was injected using an 80 °C injector and operated in splitless mode, with high-purity helium (He) as the carrier gas at a flow rate of 1.0 mL/min. The sample was analysed using a DB-FFAP column (30 m × 250 μm × 0.25 μm, Agilent) with an initial temperature of 50 °C (held for 1 min), ramped at 7 °C/min to 200 °C, and held for 1 min. Mass spectrometry was performed with electron ionisation (EI) at −70 eV, an ion source temperature of 250 °C, a transfer line temperature of 280 °C, and an electron multiplier voltage of 0.95 kV. Data were collected in the full-scan mode over a mass-to-charge ratio range of m/z 33–200.

### Transcriptomic sequencing

Transcriptomic sequencing was conducted as previously reported [34]. Total RNA was extracted from the tumours using the TRIzol reagent (Takara, Shiga, Japan). Approximately 60 mg of tissue was powdered in liquid nitrogen, homogenised, and centrifuged to isolate RNA. Chloroform/isoamyl alcohol was used to extract RNA, and was precipitated using isopropyl alcohol. Purified RNA was quantified, and its quality was assessed using NanoDrop and Agilent 2100 (Thermo Fisher). Oligo(dT)-attached beads were used to isolate mRNA, which was fragmented and reverse-transcribed into cDNA. cDNA was purified, end-repaired, and ligated with adaptors for PCR amplification. The quality and quantity of the cDNA library were checked using Agilent 2100 and qPCR. The library was treated with duplex-specific nuclease, flow-cell amplified, and single-end sequenced on an Illumina NovaSeq 6000 to obtain high-quality sequencing data (MajorBio, Shanghai, China).

### RT-qPCR

Samples consisting of 0.1 g of tumour were homogenised in 1 mL of TRIzol reagent (Takara). RNA was extracted using the chloroform-isopropanol method. RNA from tissues was reverse-transcribed into cDNA using Evo M-MLV RT Master Mix (AG, Guangzhou, China) according to the manufacturer’s instructions. Subsequently, mRNA expression was analysed using a SYBR ® Green Premix Pro Taq HS qPCR Kit (Rox Plus) (AG) with a LightCycler 96 detection system (Roche, Basel, Switzerland), and β-actin was used for normalisation. The primer sequences are listed in Supplementary Table 1.

### Western blotting

Total protein from the tumours was extracted using RIPA buffer (Beyotime), lysed from tissues with the addition of protease inhibitors (1:100, CWBIO, Beijing, China) and phosphatase inhibitors (1:100, CWBIO), separated by 10% sodium dodecyl sulfate-polyacrylamide gel electrophoresis, and transferred onto polyvinylidene difluoride membranes. After 16 h incubation with primary antibodies (listed in Supplementary Table 2) at 4 °C, the membranes were washed and incubated with HRP-conjugated secondary antibodies (Proteintech, Wuhan, China) for 1.5 h at 4 °C. Membranes were washed and visualised using enhanced chemiluminescence (Biosharp, Anhui, China).

### ELISA

Tumours in each group were ground in 100 μL of PBS per 0.1 g, followed by centrifugation for 10 min at 4000 × *g* and 4 °C. Cxcr6 and Ccl5 levels in the tissues were measured using ELISA kits (Neobioscience, Shenzhen, China), following the manufacturer’s instructions.

### Bioinformatic analysis

Transcription factor-binding sites for Pkonx2, Irf4, Cebpb, and Meox1 in the promoter regions of Cxcr6 and Ccl5 were predicted using the hTFtarget database (http://bioinfo.life.hust.edu.cn/hTFtarget#!/) [39]. The correlation between MEOX1 and CXCR6 or CCL5, effector T cell and MEOX1, CXCR6, or CCL5 were determined using the GEPIA2 database (http://gepia2.cancer-pku.cn/#correlation) [40].

### Dual luciferase assay

Dual luciferase assay was conducted using Dual-Luciferase® Reporter Assay System kit (E2920, Promega, Madison, WI, USA). CT26 cells were seeded into a 96-well plate (Nest, China) and co-transfected with the Meox1 overexpression plasmid, pGL3.1-Cxcr6 promoter luciferase reporter plasmid or pGL3.1-Ccl5 promoter luciferase reporter plasmid and the Renilla plasmid using the Lipo3000 kit and Opti-MEM medium (Gibco). Replace the Opti-MEM medium with 10%FBS DMEM medium (Gibco) after 4 h of transfection. After 48 h, Dual-Glo® Luciferase Assay Reagent was added to the wells and incubated at 25 °C for 1 h. Firefly luminescence was then measured with the BioTek Synergy Neo2 Multifunctional Microplate Detection Instrument. Dual-Glo® Stop & Glo® Reagent was added to the wells, and after another 1 h incubation at 25 °C, Renilla luminescence was measured. The firefly to Renilla luciferase ratio was calculated for each well and normalised using the ratio from control wells.

### CUT&Tag-qPCR

CUT&Tag-qPCR was conducted using the Hyperactive Universal CUT&Tag Assay Kit (TD904, Nanjing, Vazyme) in accordance with the manufacturer’s protocol. CT26 cells, treated or untreated with 100 mM sodium propionate, were harvested and counted. A total of 100,000 cells were transferred to an 8-well tube. After centrifugation at 600 × *g* for 5 min, the pellet was resuspended in 500 μL wash buffer. Following the removal of the supernatant, the cells were resuspended in 100 μL wash buffer. One hundred microliters of this suspension were transferred to an 8-well tube containing activated ConA Beads Pro and incubated at room temperature with inversion for 10 min. The tube was placed on a magnetic rack to remove the supernatant. Fifty microliters of antibody buffer were added to resuspend the cell-bead complex, followed by 5 μg of recombinant Anti-Histone H3 (acetyl K14) antibody (ab52946, Abcam, Cambridge, UK). The mixture was incubated overnight at 4 °C. After incubation, the tube was placed on the magnetic rack to remove the supernatant, and 50 μL of secondary antibody diluted in Dig-wash Buffer was added and incubated at room temperature for 1 h. The cells were washed three times with 200 μL Dig-wash Buffer. Next, 100 μL of diluted pA/G-Tnp Pro was added and incubated at room temperature for 1 h. The cells were washed again with 200 μL Dig-wash Buffer. Fifty microliters of TTBL was added and incubated at 37 °C for 1 h. Two microliters of 10% SDS and 1 pg of DNA Spike-in were added, mixed, and incubated at 55 °C for 10 min. The supernatant was carefully transferred to a new 8-well tube, discarding the beads. Fifty microliters of DNA Extract Beads Pro were added and incubated with inversion at room temperature for 20 min. The tube was placed on the magnetic rack to remove the supernatant, and 200 μL of 1× B&W Buffer was added and incubated for 30 s. The supernatant was carefully removed, and the tube was left open at room temperature for 5 min until dry. Finally, 15 μL of ddH2O and 5 μL of Stop Buffer were added, mixed, and incubated at 95 °C for 5 min. The tube was placed on the magnetic rack to collect the product for qPCR analysis. The primer sequences used are listed in Supplementary Table 1. ΔCT values for each group were calculated using DNA Spike-in as the internal reference. The calculations were performed as follows:

For the experimental group: ΔCT_treatment_ = CT_treatment_ − CT_DNA Spike-in_.

For the control group: ΔCT_control_ = CT_control_ − CT_DNA Spike-in_.

### Statistical analysis

Data were tested for normality and variation before statistical tests were applied. Data were analysed using Student’s unpaired *t*-test, multiple *t*-test or two-way ANOVA with GraphPad Prism 9. The correlation study was analysed using a Spearman rank correlation test. The number of asterisks represents the degree of significance with respect to *p* values, with the latter presented within each figure or figure legend. All the biochemical experiments were repeated at least three times with similar results.

## Results

### Impact of gut microbiota on the efficacy of IRT

**Fig. 1:**
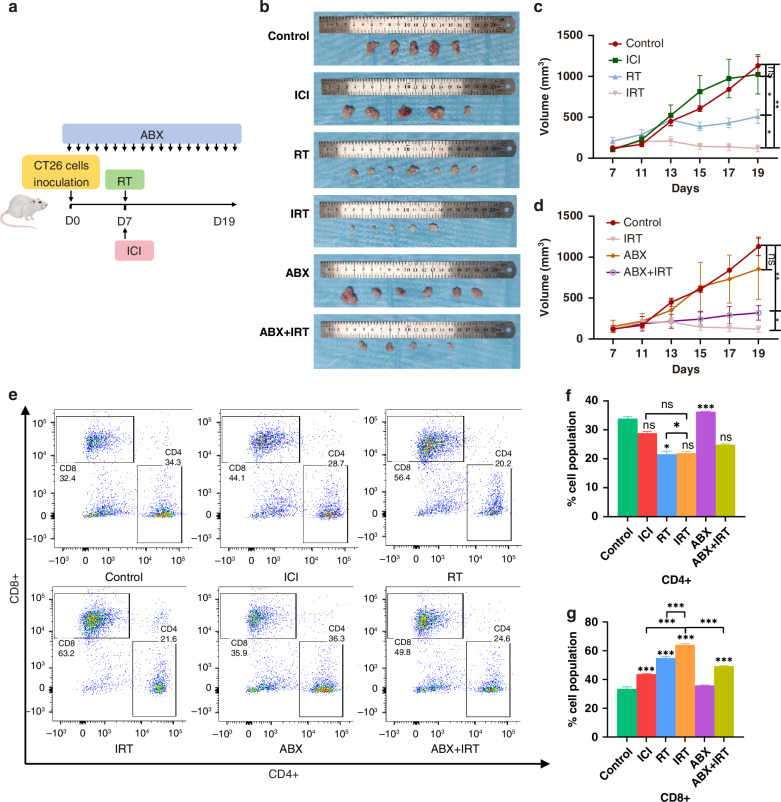
Gut Microbiota’s Impact on IRT Efficacy. **a** The protocol for in vivo experiments (more details in the “Materials and methods” section). **b** Representative gross image of subcutaneous tumour. **c** Tumour volume in mice across Control, ICI, RT and IRT groups. **d** Tumour volume in mice across Control, IRT, ABX, and ABX + IRT groups. **e** Representative contour plots of CD4^+^ T cells and CD8^+^ T cells. **f** Summary statistics of CD4^+^ T cells. **g** Summary statistics of CD8^+^ T cells. ***p < 0.001, **p < 0.01, *p < 0.05, ns not significant.

To investigate the effect of combined radiotherapy and immunotherapy compared to single treatments on MSS-CRC, we established a subcutaneous tumour model in mice (Fig. 1a). Dynamic monitoring of tumour volume revealed that immunotherapy alone had no significant therapeutic effect on tumours, whereas radiotherapy significantly reduced the tumour volume (Fig. 1b, c). The combination of radiotherapy and immunotherapy further reduced the tumour volume, indicating that combined therapy can markedly reduce the volume of MSS-CRC (Fig. 1b, c). Furthermore, dynamic body weight monitoring of the mice showed no significant differences among the groups (Supplementary Fig. 1a). To explore the effect of gut microbiota on the efficacy of combined therapy in MSS-CRC, we altered the gut microbiome in mice using four antibiotics (Fig. 1a). Dynamic monitoring of tumour volume showed that antibiotics alone had no significant effect on subcutaneous tumours; however, adding antibiotics to the combined radiotherapy and immunotherapy significantly increased tumour volume (Fig. 1b, d). This suggests that gut microbiota influences the efficacy of combined radiotherapy and immunotherapy in MSS-CRC. Additionally, dynamic body weight measurements revealed significant differences in the ABX and IRT groups compared to other groups (Supplementary Fig. 1b). Flow cytometric analysis of the tumours from each group indicated that combined radiotherapy and immunotherapy significantly increased the proportion of CD8^+^ T cells within the tumours compared to single treatments. However, no significant changes were observed in CD4^+^ T or NK cells (Fig. 1e–g, Supplementary Fig. 1c, d). Alteration of the gut microbiota with antibiotics significantly inhibited the infiltration of CD8^+^ T cells into the tumours (Fig. 1e–g, Supplementary Fig. 1c, d). In summary, the dysbiosis of gut microbiota diminishes the efficacy of combined radiotherapy and immunotherapy.

### Propionate and high-propionate-producing bacteria correlate with enhanced efficacy of IRT

**Fig. 2:**
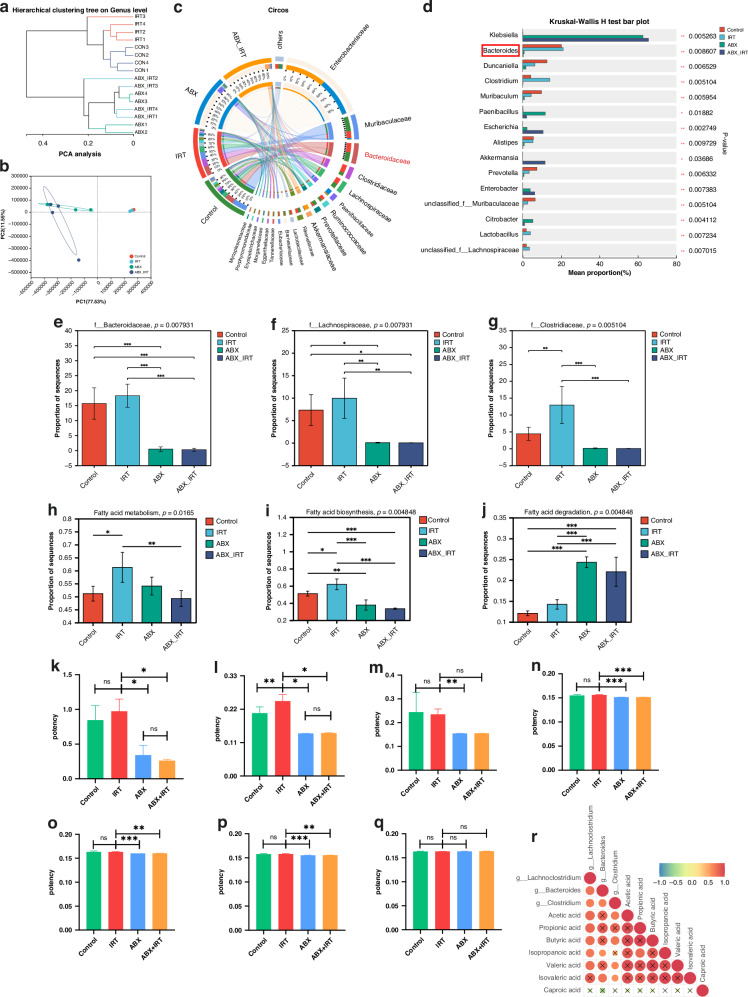
Exploration of key bacteria and bacterial metabolites affecting the efficacy of IRT. **a** Cluster tree analysis of multiple samples. **b** PCA analysis of multiple samples in metagenomics. **c** Circos plot of differential analysis across multiple groups at the family level. **d** Bar plot of differential analysis across multiple groups at the genus level. **e** Relative abundance of Bacteroidaceae. **f** Relative abundance of Lachnospiraceae. **g** Relative abundance of Clostridiaceae. **h** Relative abundance of fatty acid metabolism. **i** Relative abundance of fatty acid biosynthesis. **j** Relative abundance of fatty acid degradation. Concentration of acetic acid (**k**), propionic acid (**l**), butyric acid (**m**), isobutyric acid (**n**), valeric acid (**o**), isovaleric acid (**p**), and caproic acid (**q**) in intestinal contents. **r** Correlation analysis between SCFAs and gut bacteria. ***p < 0.001, **p < 0.01, *p < 0.05, ns not significant.

We performed a metagenomic analysis on samples from different mouse groups to investigate the gut microbiota that affects the efficacy of combined radiotherapy and immunotherapy. Hierarchical clustering analysis revealed a distinct separation in the community structure between the ABX and ABX + IRT groups compared with the other two groups, indicating that ABX significantly disrupted the gut microbiota composition (Fig. 2a). Principal component analysis (PCA) of gut microbiota β-diversity confirmed these findings (Fig. 2b). The presence of the gut microbiota at the phylum level showed clear differences among the groups (Supplementary Fig. 2a). At the family level, the differential analysis revealed that Bacteroidaceae, Muribaculaceae, Clostridiaceae, and Lachnospiraceae were significantly more abundant in the IRT group than in the other groups, whereas Enterobacteriaceae and Akkermansiaceae were more abundant in the ABX + IRT group than in the other groups (Fig. 2c). At the genus level, *Bacteroides*, *Clostridium*, and *Lactobacillus* were more abundant in the IRT group, suggesting that these genera may be associated with the efficacy of combined radiotherapy and immunotherapy (Fig. 2d). Further analysis of the abundance of Bacteroidaceae, Clostridiaceae, and Lachnospiraceae families in each group indicated that these families may be related to the therapeutic efficacy of the combined treatment (Fig. 2e–g). Among them, *Bacteroides* had the highest relative abundance, and statistical analysis at the species level revealed that they were potentially associated with the efficacy of the combined therapy (Supplementary Fig. 2b–g).

Additionally, we analysed changes in metabolic genes and metabolism in the gut bacteria using the KEGG pathway analysis. The results indicated that genes in the IRT group were significantly involved in fatty acid metabolism and fatty acid biosynthesis, whereas the ABX-treated groups were involved in fatty acid degradation (Fig. 2h–j). Fatty acid metabolism in the gut involves long-chain and short-chain fatty acid metabolism. We explored the alteration of long-chain fatty acid metabolism across the groups using nontargeted metabolomics. Hierarchical clustering analysis and PCA revealed a distinct separation in the community structure among the four groups (Supplementary Fig. 2h, i). No significant changes in long-chain fatty acid metabolism were observed, with only three long-chain fatty acids showing altered abundance, which did not correlate with the tumour volume (Supplementary Fig. 2j–l); therefore, we focused on SCFAs. GC-MS analysis of SCFAs in the intestinal contents of the four mouse groups revealed a significant increase in propionate levels in the IRT group, whereas ABX treatment markedly reduced its abundance (Fig. 2k–q). Correlation analysis between SCFAs and *Bacteroides*, *Clostridium*, and *Lachnoclostridium* revealed a significant production of SCFAs, including propionate, showing a significant positive correlation between propionate and *Bacteroides* (Fig. 2r). These results suggest that propionate- and high-propionate-producing *Bacteroides* are closely associated with the efficacy of combined radiotherapy and immunotherapy.

### Propionate and propionate-high-producing *B. fragilis* sensitise tumours to IRT

**Fig. 3:**
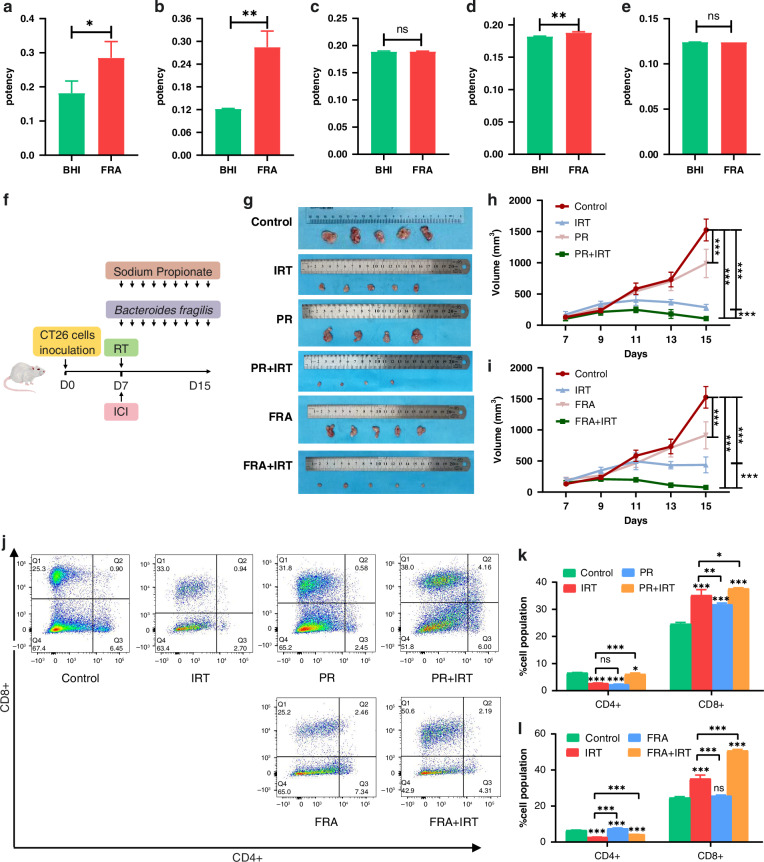
Sensitisation to IRT by propionate and propionate-producing *B. fragilis.* Concentration of acetic acid (**a**), propionic acid (**b**), butyric acid (**c**), isobutyric acid (**d**), valeric acid (**e**) in *B. fragilis* culture supernatant. **f** The protocol for in vivo experiments (more details in the “Materials and methods” section). **g** Representative gross image of subcutaneous tumour. **h** Tumour volume in mice across Control, IRT, PR, and PR + IRT groups. **i** Tumour volume in mice across Control, IRT, FRA, and FRA + IRT groups. **j** Representative contour plots of CD4^+^ T cells and CD8^+^ T cells. **k** Summary statistics of CD4^+^ T cells and CD8^+^ T cells in mice across Control, IRT, PR, and PR + IRT groups. **l** Summary statistics of CD4^+^ T cells and CD8^+^ T cells in mice across Control, IRT, FRA, and FRA + IRT groups. ***p < 0.001, **p < 0.01, *p < 0.05, ns not significant.

In the current study, SCFA production was assessed by collecting culture supernatants from *B. fragilis*. The results demonstrated that *B. fragilis* produced acetate, propionate, and isobutyrate (Fig. 3a–e). Based on these findings, we explored the influence of propionate and *B. fragilis* on the efficacy of combined radiotherapy and immunotherapy in vivo (Fig. 3f). Dynamic monitoring of subcutaneous tumour volumes in mice revealed that propionate alone inhibited tumour growth. The combination of propionate with radiotherapy and immunotherapy significantly reduced tumour volumes than did radiotherapy and immunotherapy alone (Fig. 3g, h). Body weight monitoring of the mice indicated that propionate did not affect radiotherapy or immunotherapy-induced weight changes (Supplementary Fig. 3a). Similarly, *B. fragilis* alone exhibited tumour-inhibitory effects; however, when combined with radiotherapy and immunotherapy, this bacterium significantly reduced tumour volumes compared to combined radiotherapy and immunotherapy alone (Fig. 3g, i). Body weight measurements showed that adding *B. fragilis* did not affect weight changes caused by radiotherapy and immunotherapy (Supplementary Fig. 3b). Further tumour analysis by flow cytometry revealed that the proportion of CD8^+^ T cell infiltration was significantly higher in the PR + IRT and FRA + IRT groups than in the IRT group (Fig. 3j–l). Additionally, CD8^+^ T cell cytotoxicity, as indicated by elevated levels of Ifng, IL-1β, and Tnf, was notably increased (Supplementary Fig. 3c–e). The addition of propionate and *B. fragilis* did not significantly impact the effect of radiotherapy and immunotherapy on CD4 + T and NK cells (Fig. 3j–l, Supplementary Fig. 3f–h). Overall, propionate and *B. fragilis* enhanced the efficacy of combined radiotherapy and immunotherapy.

### Propionate and *B. fragilis* enhance CD8^+^ T cell infiltration

**Fig. 4:**
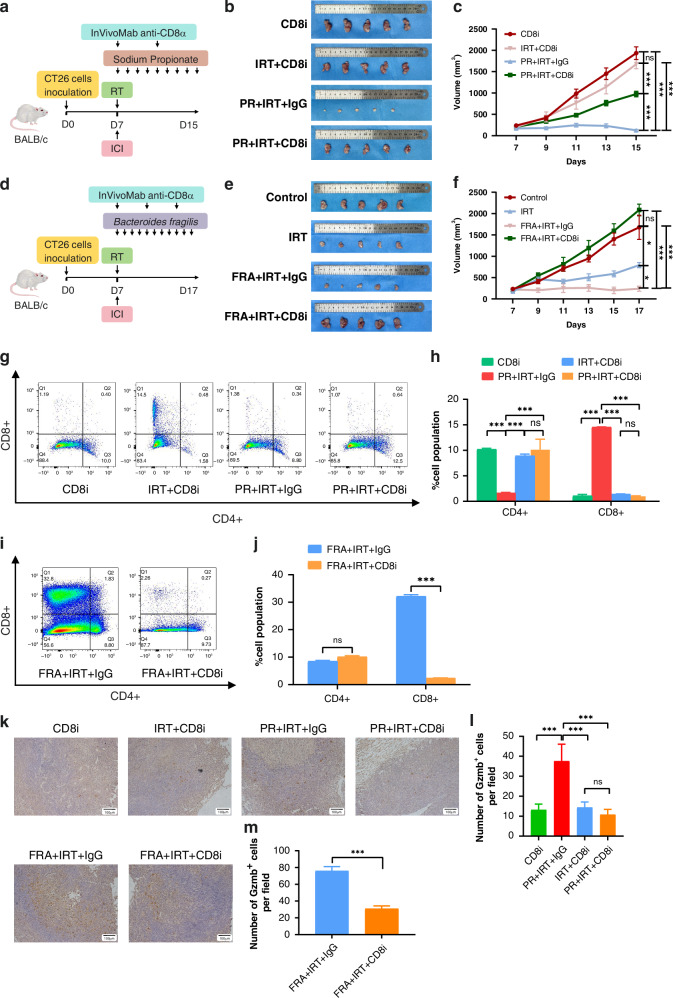
Propionate and *B. fragilis* enhance CD8+ T Cell infiltration and sensitisation to therapy. **a** The protocol for in vivo experiments (more details in the “Materials and methods” section). **b** Representative gross image of subcutaneous tumour. **c** Tumour volume in mice across CD8i, IRT+CD8i, PR + IRT+IgG, and PR + IRT+CD8i groups. **d** The protocol for in vivo experiments (more details in the “Materials and methods” section). **e** Representative gross image of subcutaneous tumour. **f** Tumour volume in mice across Control, IRT, FRA + IRT+IgG, and FRA + IRT+CD8i groups. **g** Representative contour plots of CD4^+^ T cells and CD8^+^ T cells. **h** Summary statistics of CD4^+^ T cells and CD8^+^ T cells in mice across CD8i, IRT+CD8i, PR + IRT+IgG, and PR + IRT+CD8i groups. **i** Representative contour plots of CD4^+^ T cells and CD8^+^ T cells. **j** Summary statistics of CD4^+^ T cells and CD8^+^ T cells in mice between FRA + IRT+IgG and FRA + IRT+CD8i. **k** Representative immunohistochemical images. **l** Quantitative analysis of Gzmb^+^ cells per field of view. **m** Quantitative analysis of Gzmb+ cells per field of view between FRA + IRT+IgG and FRA + IRT+CD8i groups. ***p < 0.001, **p < 0.01, *p < 0.05, ns not significant.

We performed a rescue experiment using in vivo anti-CD8 antibody to investigate the regulatory effects of propionate on intratumoral CD8^+^ T cells (Fig. 4a). InVivoMab anti-mouse CD8α significantly inhibited the tumour-reducing efficacy of IRT and negated the sensitising effect of propionate on combined radiotherapy and immunotherapy (Fig. 4b, c). Mouse body weight monitoring showed that InVivoMab anti-mouse CD8α did not affect the weight of the mice (Supplementary Fig. 4a). To explore the effect of *B. fragilis* on intratumoral CD8^+^ T cells, we conducted a similar rescue experiment (Fig. 4d). Results showed that InVivoMab anti-mouse CD8α significantly diminished the sensitising effect of *B. fragilis* on combined radiotherapy and immunotherapy (Fig. 4e, f). Body weight measurements indicated that the antibody did not affect the mice’s weight (Supplementary Fig. 4b). Flow cytometry revealed that the anti-CD8 antibody significantly reduced the proportion of CD8^+^ T cell infiltration in the IRT+CD8i and PR + IRT+CD8i groups; however, the CD4^+^ T cells and NK cells were unaffected (Fig. 4g, h, Supplementary Fig. 4c, d). Similarly, the anti-CD8 antibody significantly reduced the proportion of CD8^+^ T cells in the FRA + IRT+CD8i group without affecting CD4^+^ T and NK cells (Fig. 4i, j, Supplementary Fig. 4e, f). IHC staining of tumour tissues for GzmB revealed that the number of GzmB^+^ cells exhibited a consistent trend with the CD8^+^ T cell counts quantified using flow cytometry (Fig. 4k–m). In summary, the sensitising effects of propionate and *B. fragilis* on the efficacy of combined radiotherapy and immunotherapy were mediated by CD8^+^ T cells.

### Propionate enhances CD8^+^ T cell infiltration into tumours by increasing H3K14 acetylation and promoting the Meox1-Cxcr6/Ccl5 axis

**Fig. 5:**
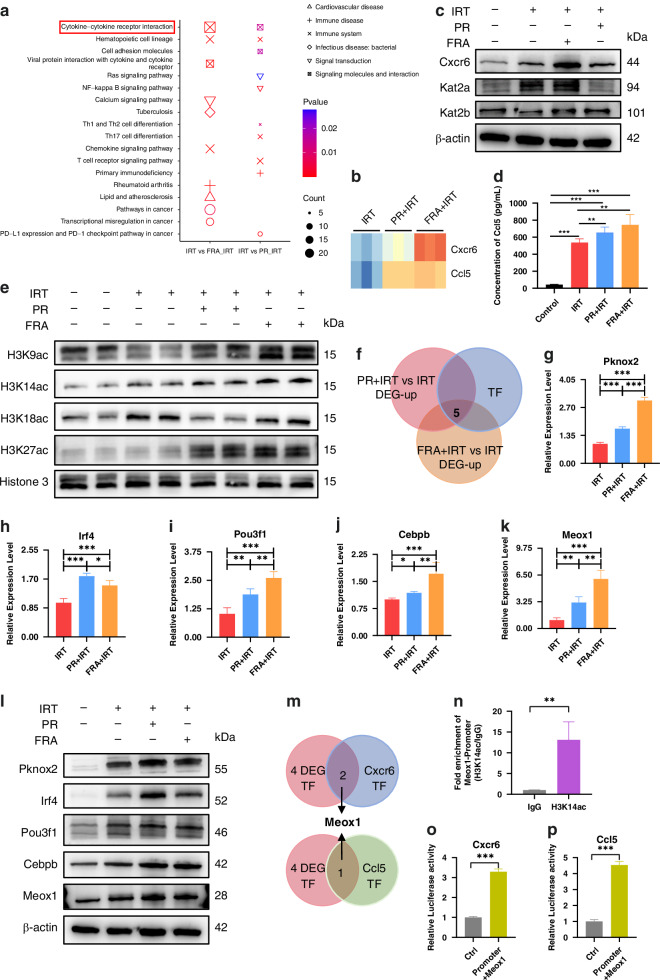
Propionate enhances CD8 + T cell infiltration via H3K14 acetylation and Meox1-Cxcr6/Ccl5 axis. **a** KEGG pathway enrichment of differentially expressed genes (DEGs). **b** The mRNA level of Cxcr6 and Ccl5 in RNA sequencing. **c** The protein level of Cxcr6, Kat2a and Kat2b in tumour tissue. **d** The protein level of Ccl5 in tumour tissue. **e** Histone acetylation levels associated with SCFAs. **f** Intersection analysis of upregulated DEGs and Transcription Factors (TF)s. The relative mRNA level of Pknox2 (**g**), Irf4 (**h**), Pou3f1 (**i**), Cebpb (**j**) and Meox1 (**k**) in tumour tissue. **l** The protein level of Pknox2, Irf4, Pou3f1, Cebpb and Meox1 in tumour tissue. **m** Intersection analysis of upregulated TFs with those regulating Cxcr6 and Ccl5. **n** qPCR results of H3K14ac on the Meox1 promoter following CUT&Tag. **o** Dual-Luciferase reporter assay results of Meox1 binding to the Cxcr6 promoter. **p** Dual-Luciferase reporter assay results of Meox1 binding to the Ccl5 promoter. ***p < 0.001, **p <0.01, *p < 0.05.

We investigated the molecular mechanisms underlying the effects of propionate by RNA sequencing of tumours from the IRT, PR + IRT, and FRA + IRT groups. Cluster analysis showed distinct gene expression profiles among the groups, with the PR + IRT and FRA + IRT groups showing similar expression patterns (Supplementary Fig. 5a). Differential expression gene (DEG) analysis and KEGG pathway enrichment revealed that the cytokine-cytokine receptor interaction pathway was the most significantly altered (Supplementary Fig. 5b, Fig. 5a). Notably, Cxcr6 and Ccl5 were identified as intersecting DEGs within this pathway, and their mRNA expression levels were significantly higher in the PR + IRT and FRA + IRT groups than in the IRT group (Fig. 5b). Western blotting and ELISA confirmed that the protein levels of Cxcr6 and Ccl5 were also significantly elevated in tumours from the PR + IRT and FRA + IRT groups (Fig. 5c, d). Additionally, in vitro experiments using CT26 cells confirmed the higher transcriptional levels of Cxcr6 and Ccl5 in the PR + IRT and FRA + IRT groups (Supplementary Fig. 5c, d).

We assessed histone acetylation modifications in tumours as the core mechanism of action of propionate. We found that propionate and *B. fragilis* primarily mediated the acetylation of H3K14, and the key acetyltransferase responsible for this modification was Kat2a (Fig. 5c, e). As propionate and *B. fragilis* upregulate Cxcr6 and Ccl5 transcription, we hypothesised that these genes may undergo direct H3K14 acetylation. However, CUT&Tag-qPCR results indicated that the promoter regions of Cxcr6 and Ccl5 showed no H3K14 acetylation following propionate treatment (Supplementary Fig. 5e, f). As transcription factors are crucial in gene transcription regulation, we analysed the DEGs for transcription factors and identified five genes that were significantly upregulated following propionate and *B. fragilis* treatment (Fig. 5f). qPCR analysis of tumours from each group revealed that Pknox2, Irf4, Pou3f1, Cebpb, and Meox1 were significantly upregulated by propionate and *B. fragilis* (Fig. 5g–k). Western blot analysis confirmed that the protein levels of these transcription factors were significantly increased (Fig. 5l). Further bioinformatic analysis validated the binding of these transcription factors to Cxcr6 and Ccl5 promoters (Fig. 5m). Meox1 was found to bind to Cxcr6 and Ccl5 promoters (Fig. 5m, Supplementary Fig. 5g, h). CUT&Tag-qPCR confirmed the H3K14 acetylation in the Meox1 promoter region (Fig. 5n). Dual-luciferase reporter assays demonstrated that Meox1 could bind to Cxcr6 and Ccl5 promoters and enhance their transcription (Fig. 5o, p). Additionally, correlation analysis of clinical specimens from the Cancer Genome Atlas (TCGA) database revealed significant positive correlations between MEOX1, CXCR6, CCL5, and intratumoral CD8^+^ T cells (Supplementary Fig. 6a–c), as well as between MEOX1, CXCR6, and CCL5 (Supplementary Fig. 6d, e).

### In vivo validation of propionate-mediated CD8^+^ T cell infiltration via the Meox1-Cxcr6/Ccl5 axis

**Fig. 6:**
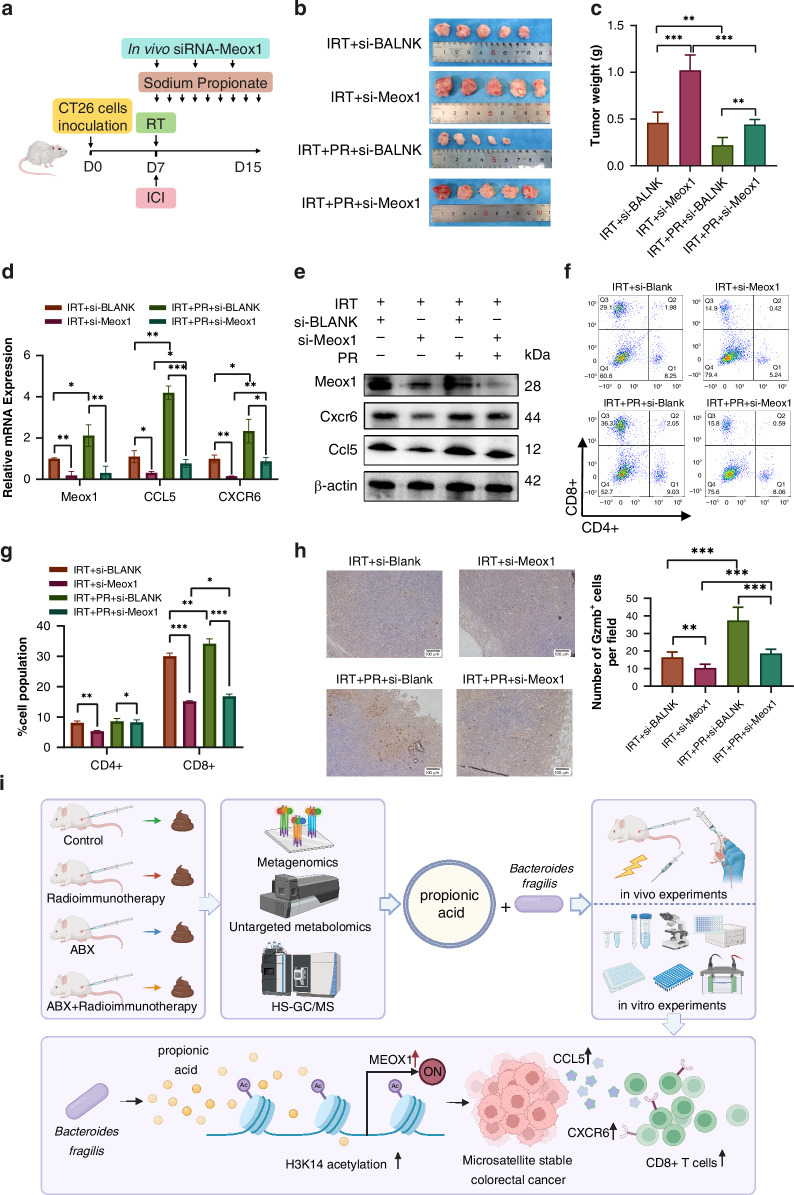
Meox1 mediates propionic acid-induced immunomodulation in vivo. **a** The protocol for in vivo experiments (more details in “Materials and methods” section). **b** Representative gross image of subcutaneous tumour. **c** Tumour weight in mice across different groups. **d** The mRNA level of Meox1, Cxcr6 and Ccl5 in tumour tissue. **e** The protein level of Meox1, Cxcr6 and Ccl5 in tumour tissue. **f** Representative contour plots of CD4^+^ and CD8^+^ T cells. **g** Summary statistics of CD4+ and CD8^+^ T cells in mice across different groups. **h** Representative immunohistochemical images and quantitative analysis of Gzmb^+^ cells per field of view. **i** Schematic diagram of this research. ***p < 0.001, **p < 0.01, *p < 0.05.

To validate the pivotal role of Meox1 in mediating the effects of propionate, we performed an in vivo rescue experiment using in vivo Meox1-targeting siRNA (Fig. 6a). Measurement of subcutaneous tumour weight revealed that intratumoral injection of Meox1 siRNA significantly attenuated propionate-induced radiosensitisation in IRT (Fig. 6b, c). Real-time qPCR analysis of tumour tissues revealed that propionate treatment significantly upregulated the transcriptional levels of Meox1, Cxcr6, and Ccl5, whereas Meox1-siRNA administration abolished these effects (Fig. 6d). Western blotting further confirmed corresponding changes in the protein abundance of these targets (Fig. 6e). Flow cytometry showed that propionate significantly increased tumour-infiltrating CD8^+^ T cells, an effect reversed by Meox1 siRNA (Fig. 6f, g). Consistent with this, IHC staining for GzmB revealed elevated GzmB^+^ cell numbers in propionate-treated tumours, which were significantly reduced upon Meox1 knockdown (Fig. 6h). These results establish that the synergistic enhancement of combined radiotherapy and immunotherapy by propionate is mechanistically dependent on Meox1.

Overall, propionate and *B. fragilis* increased H3K14 acetylation in the Meox1 promoter, boosting Meox1 transcription and protein levels. This upregulates the Meox1-Cxcr6/Ccl5 axis, enhances CD8^+^ T cell infiltration in tumours, and sensitises MSS-CRC to combined radiotherapy and immunotherapy (Fig. 6i).

## Discussion

Our study introduced neoadjuvant radiotherapy and immunotherapy as a novel approach for treating MSS-CRC. We found that the gut microbiota and its metabolites, particularly propionate and *B. fragilis*, significantly enhanced the effects of combined radiotherapy and immunotherapy by increasing the acetylation of histone H3K14, which upregulates the Meox1-Cxcr6/Ccl5 signalling axis and promotes CD8^+^ T cell activation. These findings revealed a new strategy to improve combined radiotherapy and immunotherapy via modulation of gut microbiota. Our data support the use of propionate or probiotics (particularly those meticulously screened and optimised in the second generation with high-propionate production) as adjunctive therapy, thereby broadening cancer treatment options and enhancing the efficacy of combined radiotherapy and immunotherapy.

Studies using a CT26 subcutaneous tumour mouse model revealed that immunotherapy alone was ineffective against CT26 tumours. This finding is consistent with previous reports highlighting the limited efficacy of immunotherapy in MSS-CRC [1, 7]. However, we found that combined immunotherapy and radiotherapy significantly reduced the tumour volume and markedly increased the number of intratumoral CD8^+^ T cells. These results support earlier reports indicating that radiotherapy can enhance immune system activity by increasing CD8^+^ T cell infiltration and cytotoxicity in tumours [9–12].

We altered the gut microbiota in mice treated with ABX and explored the critical gut bacteria through metagenomics. We found that the gut microbiota is crucial in modulating the effectiveness of combined radiotherapy and immunotherapy, with *Bacteroides*, *Clostridium*, and *Lachnospira* identified as key regulators. This aligns with recent reports that highlighted the influence of the gut microbiota, particularly *Bacteroides*, *Clostridium*, and *Lachnospira*, on neoadjuvant radiochemotherapy and immunotherapy [41, 42]. Reportedly, SCFAs-producing bacteria can predict the efficacy of neoadjuvant chemoradiotherapy in patients with locally advanced rectal cancer [43]. This study provides a novel perspective to expand the clinical applications of our findings.

Further analysis using GC-MS revealed that SCFAs, particularly propionate, significantly affected the efficacy of combined radiotherapy and immunotherapy. Although the effects of propionate on various cancer treatments such as radiotherapy, chemotherapy, targeted therapy, and immunotherapy have been documented [24, 25], its specific role in enhancing the efficacy of combined radiotherapy and immunotherapy has not been explored previously. Our results demonstrated that propionate and high-propionate-producing *B. fragilis* enhanced tumour sensitivity to this combined treatment by promoting CD8^+^ T cell infiltration into tumours, and this finding is consistent with existing reports [44, 45]. However, previous studies have indicated that propionate suppresses antigen-specific CD8^+^ T cell activation by inhibiting the production of IL-12 by dendritic cells [46]. This discrepancy might arise from differences between in vitro and in vivo experiments because prior reports were predominantly based on in vitro assays, whereas our conclusions were based on in vivo findings.

Notably, we observed that *B. fragilis* had a more pronounced effect on tumour sensitisation than propionate. Our data suggest that *B. fragilis* promotes substantial CD8^+^ T cell infiltration into tumours, possibly because it produces additional bacterial metabolites or cell components, including bile acids, tryptophan derivatives, and bacterial cell wall components such as PSA, all of which reportedly exert immune-regulatory effects [47–50]. This observation necessitates further investigation, and our research group will focus on exploring these potential mechanisms in future studies.

At the molecular level, our study found that propionate increases the acetylation of histone H3K14. Although the role of SCFA metabolism in the acetylation of various histone marks (including H3K9, H3K14, H3K18, H3K27) is well-documented [51, 52], the specific enhancement of H3K14 acetylation in the Meox1 gene promoter region by propionate is a novel finding. Additionally, we found that Meox1, a classical transcription factor, regulates Cxcr6 and Ccl5. Reportedly, MEOX1 knockdown significantly affects gene expression and Treg cell suppression because it has a permissive epigenetic landscape [53]. Our findings on the role of Meox1 in regulating immune responses are a novel contribution to the literature. A previous study revealed that CXCR6 coordinates the residence of CD8^+^ T cells in the brain and limits the progression of Alzheimer’s disease in mice [54]. Additionally, CCL5 overexpression has been associated with CD8^+^ T cell infiltration in solid tumours [55], and our findings support this hypothesis.

In summary, this study provides new insights for MSS-CRC treatment and a new perspective on the interactions between tumours and the host microbiome. With advances in research and technology, these findings will improve the survival and quality of life of patients with MSS-CRC. Further exploration of the potential of combining gut microbiota modulation with other therapeutic approaches and the generalisability and specificity of these microbiome-based strategies across different cancer types will be crucial for achieving comprehensive breakthroughs in cancer therapy.

Despite the important conclusions drawn from our study, three notable limitations need to be addressed. First, our study was unable to identify the precise mechanism by which Meox1 binds to specific regions despite identifying its role in enhancing neoadjuvant radiotherapy and immunotherapy efficacy. Second, our experimental validation primarily relied on a single American Type Culture Collection (ATCC) standard strain. Although this provides preliminary evidence for our hypothesis, it may not fully reflect the diversity of the gut microbiota and individual variations in clinical settings. If validated with wild-type strains isolated from immunotherapy-responsive patients, we anticipate two potential outcomes: (1) strain-specific variations in propionate production or immunomodulatory molecules may further amplify CD8^+^ T cell infiltration through the synergistic engagement of the Meox1-Cxcr6/Ccl5 axis; (2) host-microbe co-evolutionary adaptations might enhance bacterial persistence in irradiated tumour microenvironments, sustaining H3K14 acetylation-mediated epigenetic reprogramming. We are actively working to isolate wild-type *Bacteroides* strains from patients who are responsive to neoadjuvant radiotherapy and immunotherapy to obtain more representative and clinically relevant data. Finally, all conclusions in this study are based on mouse models and cell experiments without direct clinical sample validation. We anticipate that the ongoing clinical research on neoadjuvant radiotherapy and immunotherapy in patients with MSS-CRC will provide robust and comprehensive clinical evidence to support our findings.

## Supplementary information

Supplementary Figures and Tables

## Data Availability

The metagenomic and mRNA sequencing data that support the findings of this study are available from the corresponding author upon reasonable request. We confirm that all the data in this manuscript are original, and we have access to the raw data files.
