# Supplementary material for: Impact of gut microbiome on radiotherapy and immunotherapy efficacy in microsatellite-stable colorectal cancer: role of propionic acid and B. fragilis
Source: Br J Cancer. 2025 Jul 26;133(7):956–69. doi: 10.1038/s41416-025-03105-2 (PMC12480696; doi:10.1038/s41416-025-03105-2)
Supplement: Supplementary file 1 — Supplementary Figures and Tables [file 41416_2025_3105_MOESM1_ESM.docx]

Supporting information

**Impact of Gut Microbiome on Radiotherapy and Immunotherapy Efficacy in Microsatellite Stable Colorectal Cancer: Role of Propionic Acid and *B. fragilis***

*Lu Yu et al.*

**Supplementary Figures P2-7**

**Supplementary Table P8-9**


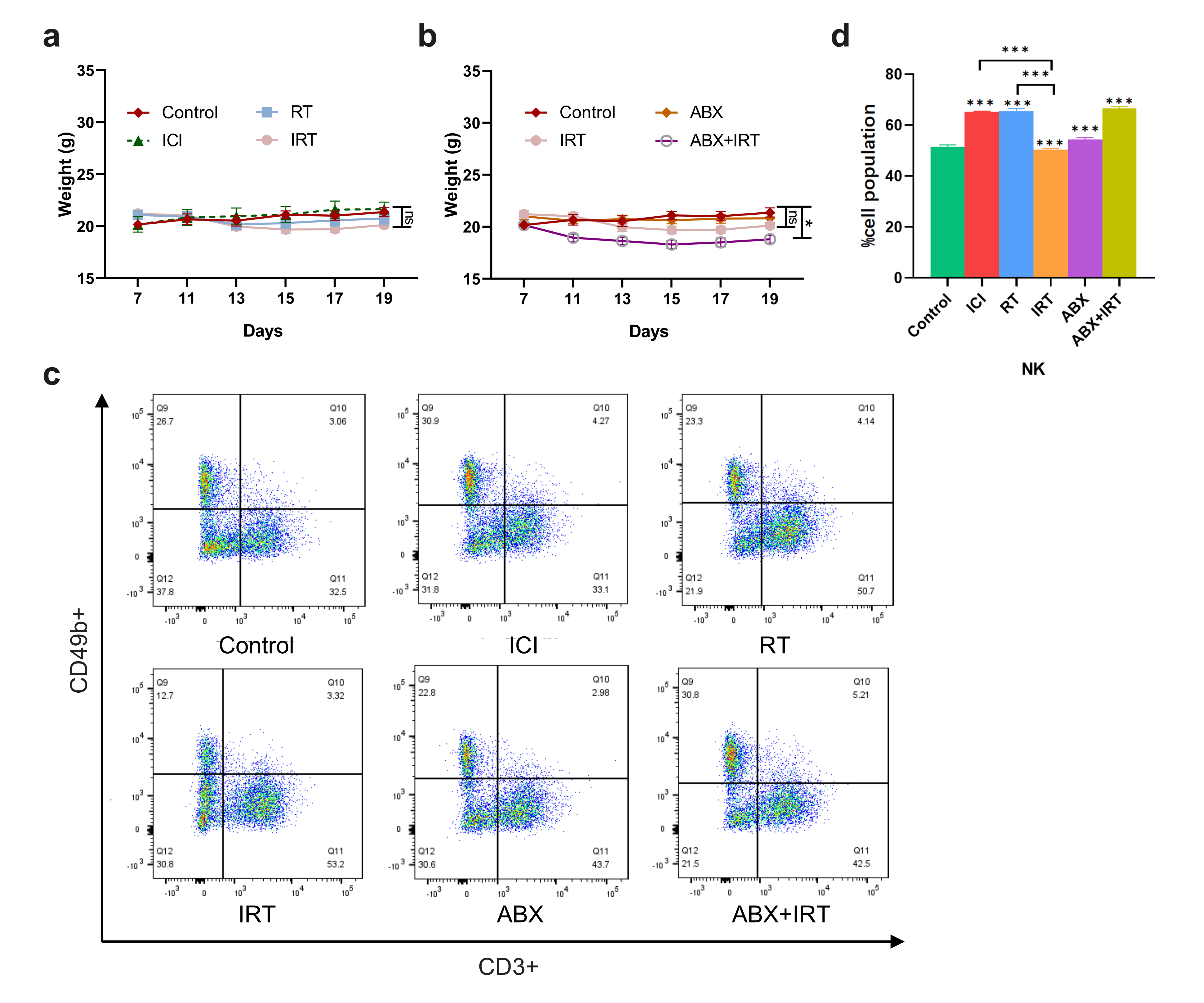


**Figure S1. Body Weight and Intratumoral NK Cell Infiltration in Mice.**

**a** Changes in body weight among mice in the Control, RT, ICI and IRT groups. **b** Changes in body weight among mice in the Control, IRT, ABX and ABX+IRT groups. **c** Representative contour plots of NK cells. **d** Summary statistics of NK cells. *** *p* < 0.001.

**
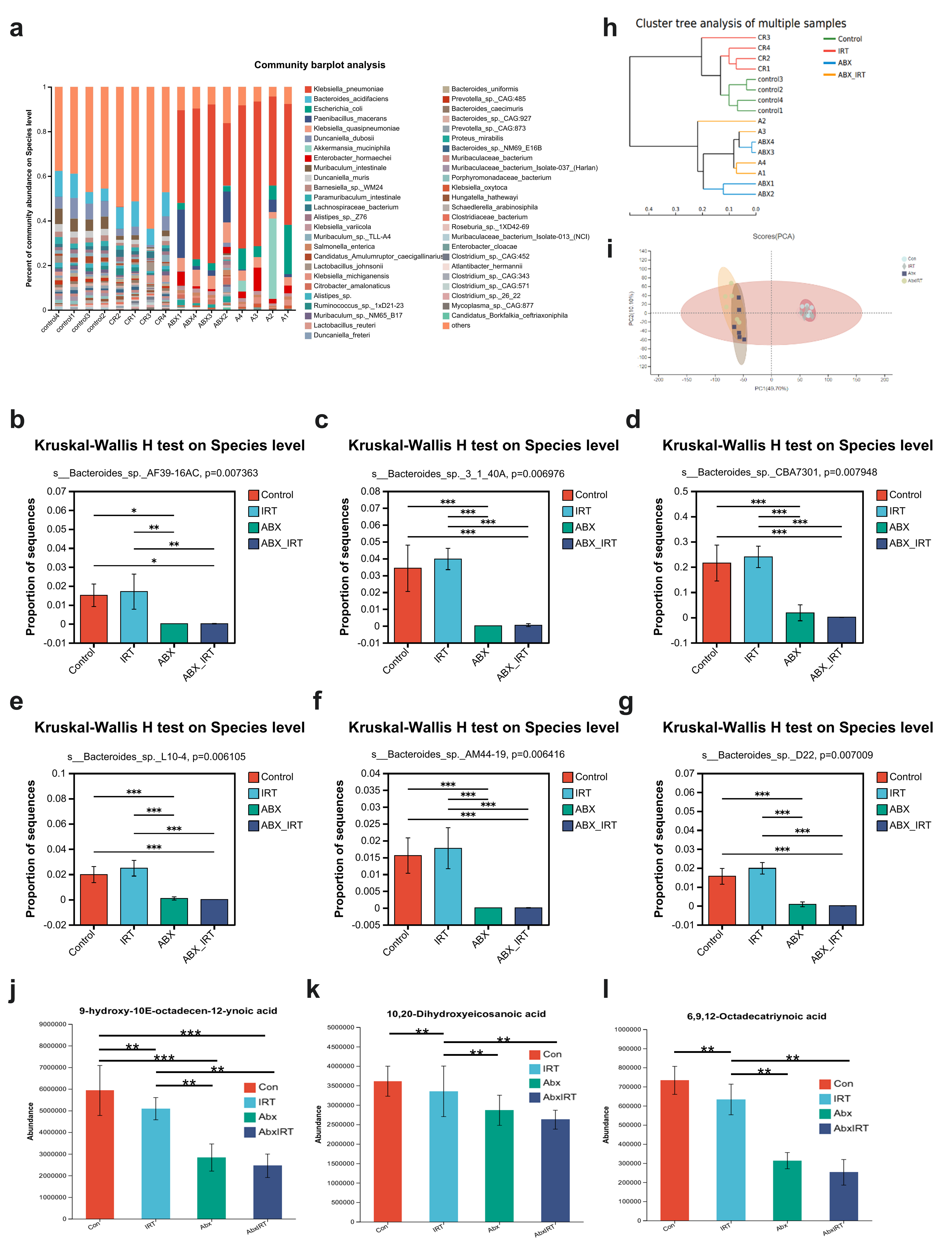
**

**Figure S2. Gut Bacteria and Their Metabolites Affect the Efficacy of Immunoradiation Therapy.**

**a** Community barplot analysis of gut microbes. **b-g** Relative abundance of different species of Bacteroides. **h** Cluster tree analysis of multiple samples in uontargeted-metabolomics. **i** PCA analysis of multiple samples. **j** Abundance of 9-hydroxy-10E-octadecen-12-ynoic acid in four groups. **k** Abundance of 10,20-Dihydroxyeicosanoic acid in four groups. **l** Abundance of 6,9,12-Octadecatriynoic acid in four groups. *** *p* < 0.001, ** *p*  < 0.01, * *p*< 0.05.

**
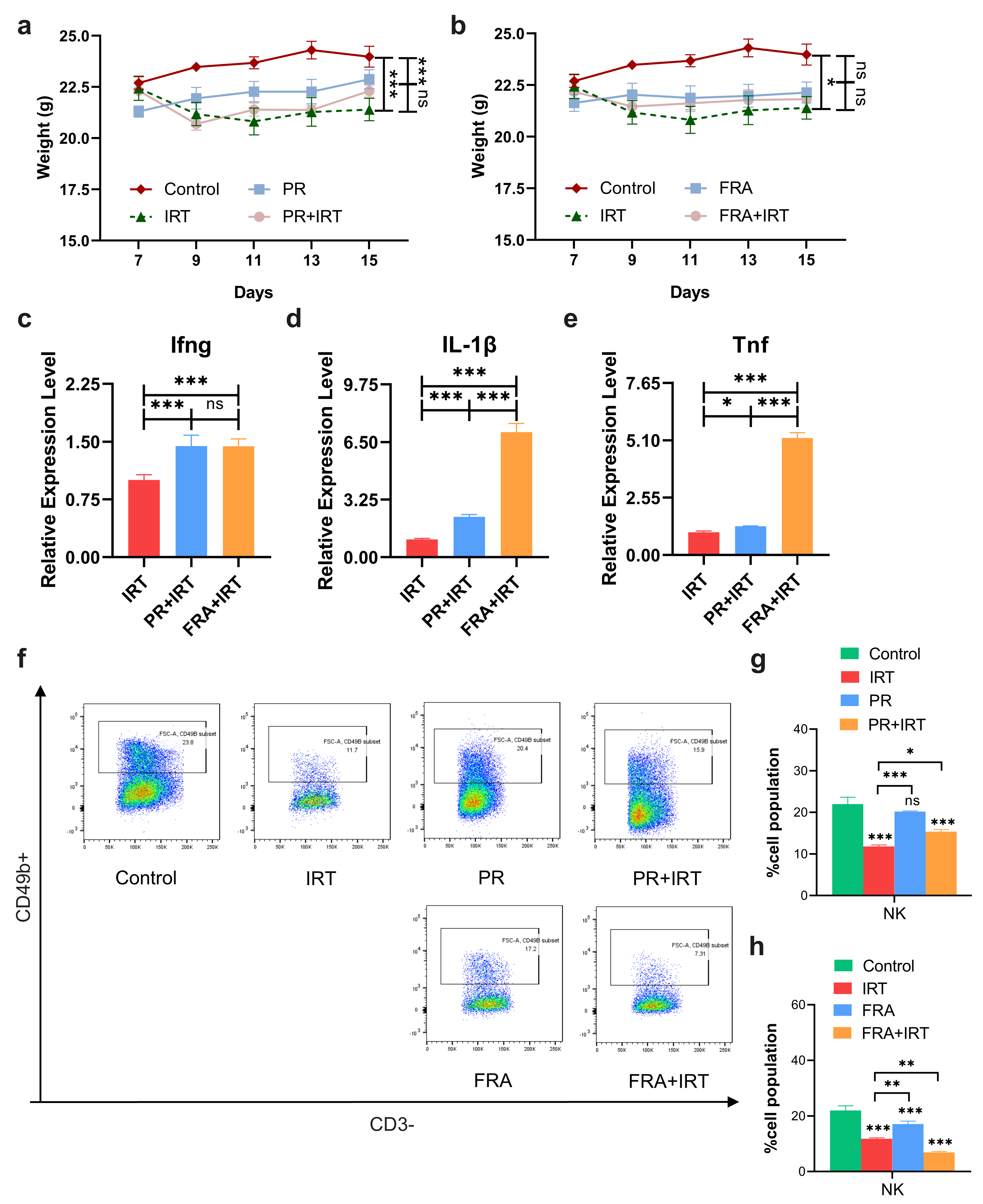
**

**Figure S3. Propionate and *Bacteroides fragilis* Enhance CD8+ Killing Ability without Influencing Body Weight or NK Cells.**

**a** Changes in body weight among mice in the Control, IRT, PR and PR+IRT groups. **b** Changes in body weight among mice in the Control, IRT, FRA and FRA+IRT groups. **c** Relative mRNA level of Ifng in tumors. **d** Relative mRNA level of IL-1β in tumors. **e** Relative mRNA level of Tnf in tumors. **f** Representative contour plots of NK cells. **g-h** Summary statistics of NK cells.*** *p* < 0.001, ** *p*  < 0.01, * *p* < 0.05.

**
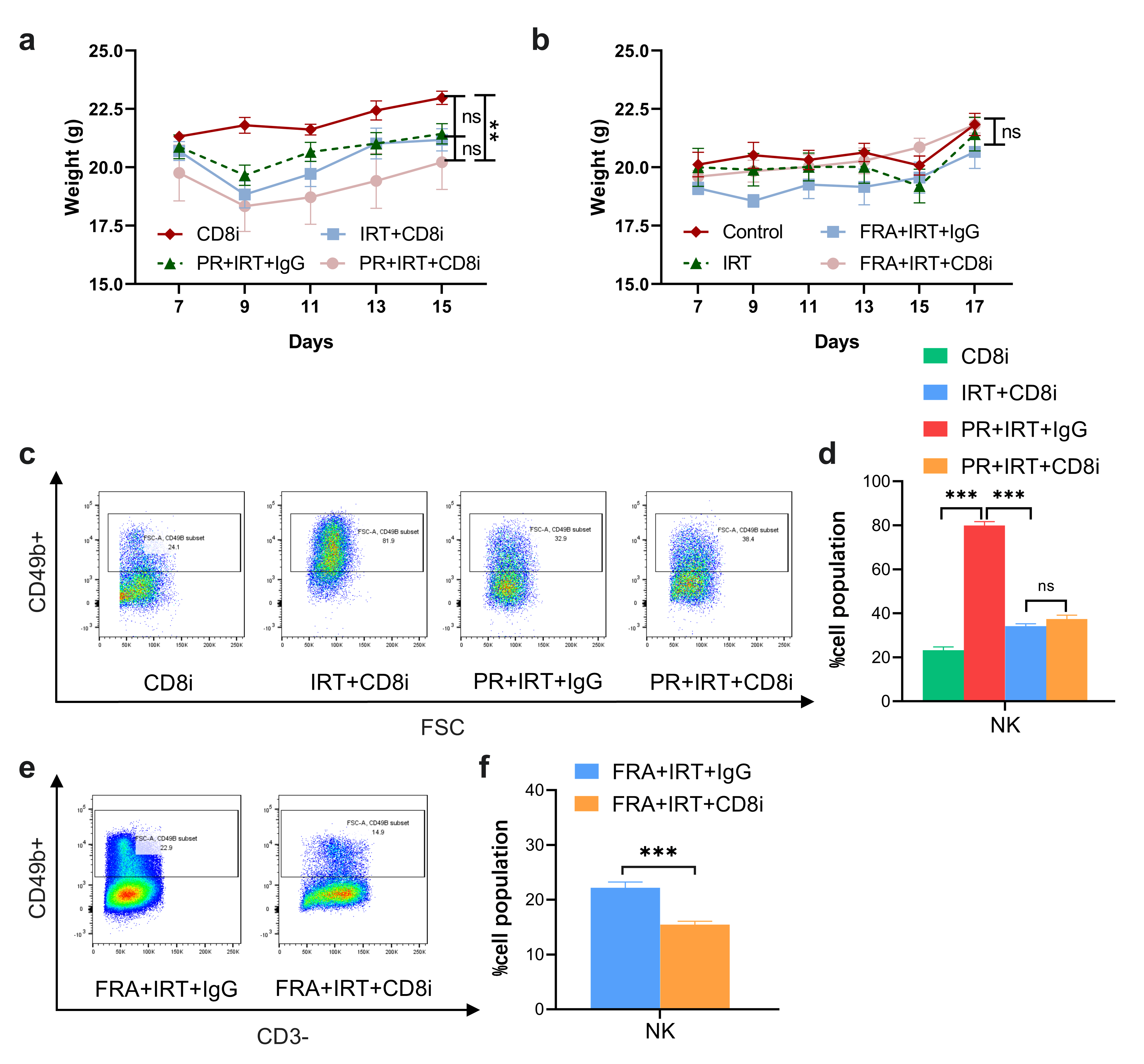
**

**Figure S4. Effect of CD8+ Inhibitors on Mouse Body Weight and NK Cells.**

**a** Changes in body weight among mice in the CD8i, IRT+CD8i, PR+IRT+IgG and PR+IRT+CD8i groups. **b** Changes in body weight among mice in the Control, IRT, FRA+IRT+IgG and FRA+IRT+CD8i groups. **c** Representative contour plots of NK cells in four groups. **d** Summary statistics of NK cells in four groups. **e** Representative contour plots of NK cells. **f** Summary statistics of NK cells. *** *p* < 0.001.

**
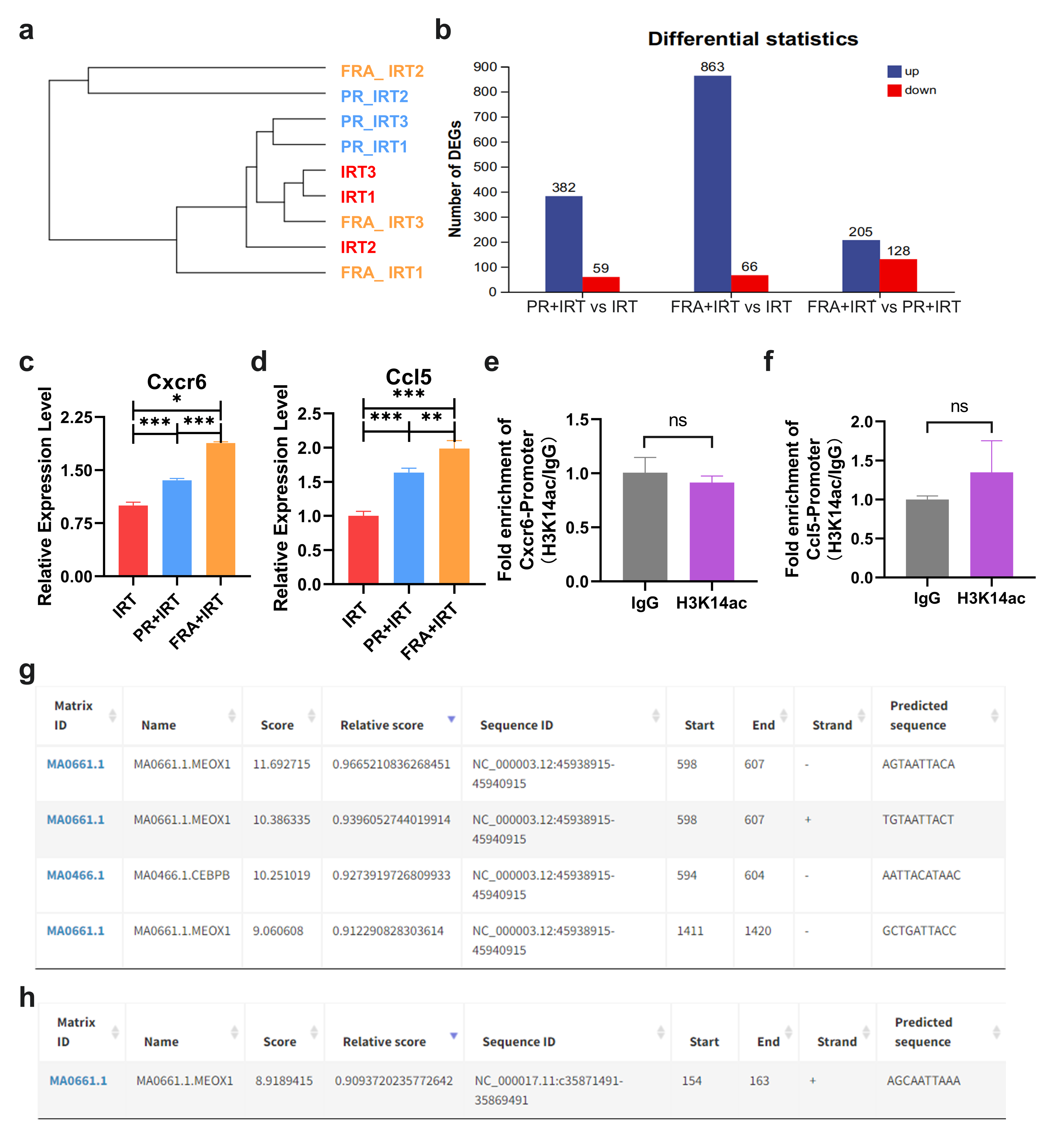
**

**Figure S5. Molecular Mechanisms of Propionate Effects IRT.**

**a** Cluster tree analysis of multiple samples in RNA sequencing. **b** Number of DEGs in differential statistics. **c** Relative mRNA level of Cxcr6 in CT26 cells. **d** Relative mRNA level of Ccl5 in CT26 cells. **e** qPCR results of H3K14ac on the Cxcr6 promoter following CUT&Tag. **f** qPCR results of H3K14ac on the Ccl5 promoter following CUT&Tag. **g** Prediction of Meox1 binding sites in the Cxcr6 promoter region. **h** Prediction of Meox1 binding sites in the Ccl5 promoter region. *** *p* < 0.001, ** *p*  < 0.01, * *p* < 0.05.

**
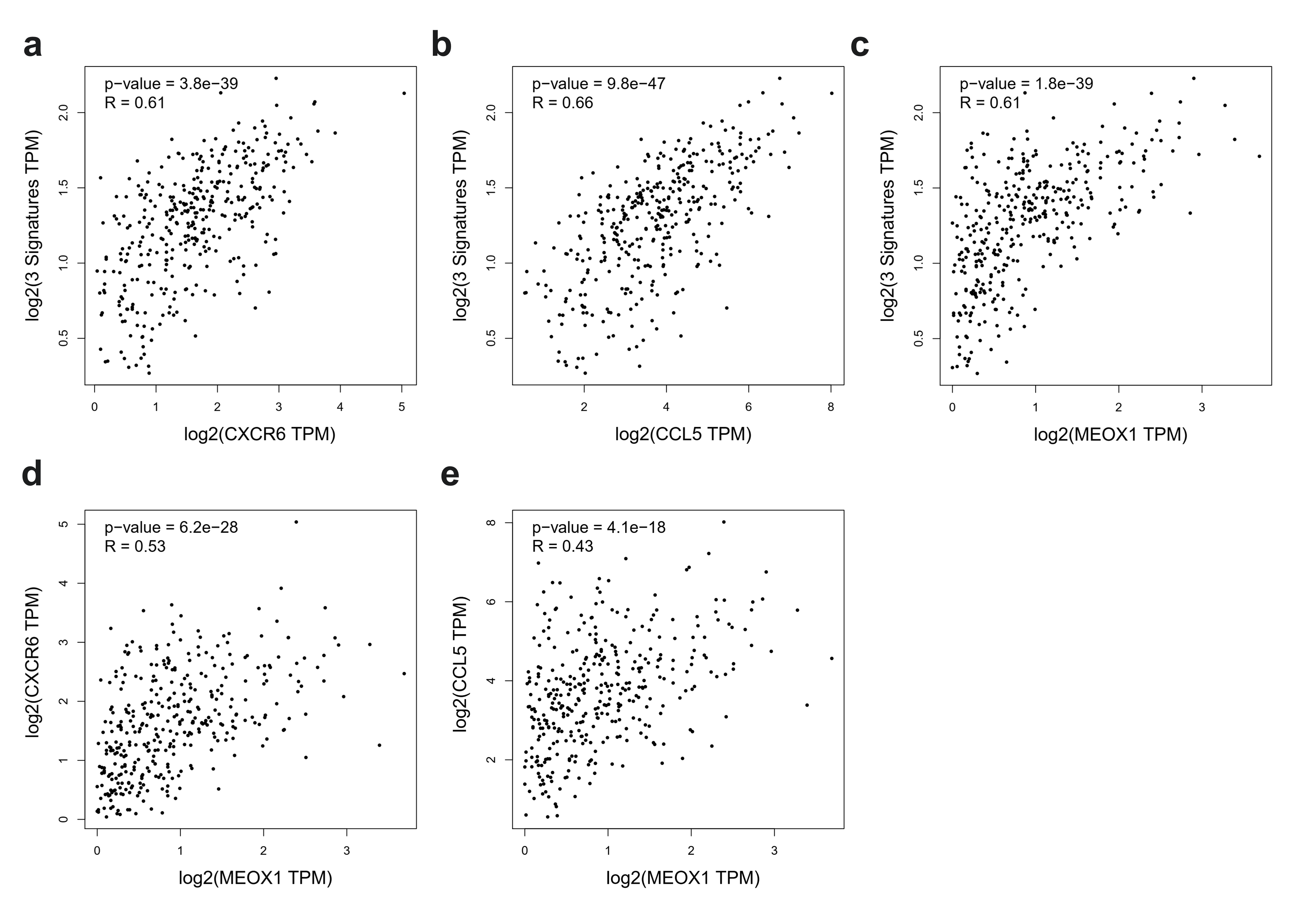
**

**Figure S6. Correlation Among Meox1, Cxcr6, CCL5, and CD8+ T Cells in COAD and READ Tumors from the GEPIA2 Database.**

**a** Correlation between CD8+ T cell and CXCR6. **b** Correlation between CD8+ T cell and CCL5. **c** Correlation between CD8+ T cell and MEOX1. **d** Correlation between MEOX1 and CXCR6. **e** Correlation between MEOX1 and CCL5.

**Supplementary Table 1. Forward and reverse primers of genes used in RT-qPCR and CUT & Tag-qPCR.**

| **Gene name** | **Primer** | **Sequence (5’-3’)** |
| --- | --- | --- |
| β-Actin | F | GTGACGTTGACATCCGTAAAGA |
|  | R | GCCGGACTCATCGTACTCC |
| Ifng | F | GCCACGGCACAGTCATTGA |
|  | R | TGCTGATGGCCTGATTGTCTT |
| IL-1β | F | GAAATGCCACCTTTTGACAGTG |
|  | R | TGGATGCTCTCATCAGGACAG |
| Tnf | F | CCTGTAGCCCACGTCGTAG |
|  | R | GGGAGTAGACAAGGTACAACCC |
| Pknox2 | F | ATCTCATGCACCCCTATCCCA |
|  | R | ACCAGTTGTTTACTTGCAGAAGG |
| Irf4 | F | AAAGGCAAGTTCCGAGAAGGG |
|  | R | CTCGACCAATTCCTCAAAGTCA |
| Pou3f1 | F | TACCGCGAAGTGCAGAAGC |
|  | R | CGTGGGTAGCCATTGAGGG |
| Cebpb | F | CAACCTGGAGACGCAGCACAAG |
|  | R | GCTTGAACAAGTTCCGCAGGGT |
| Meox1 | F | GACAGCAGCATACCCCGAC |
|  | R | CGTTGAAGATTCGCTCAGTCC |
| Cxcr6 | F | GAGTCAGCTCTGTACGATGGG |
|  | R | TCCTTGAACTTTAGGAAGCGTTT |
| Ccl5 | F | TTTGCCTACCTCTCCCTCG |
|  | R | CGACTGCAAGATTGGAGCACT |
| DNA Spike-in (CUT & Tag-qPCR) | F | AACTCTTTACCCGTCCTT |
|  | R | TGTTCCACGAATCAGC |
| Meox1 (CUT & Tag-qPCR) | F | CTTCAGGAGATGGGTAT |
|  | R | CACATTTGGGTGCTAT |

**Supplementary Table 2. Details of antibodies in Western blotting.**

| **Antibody** | **Supplier** |
| --- | --- |

| CXCR6 Antibody | DF2328, Affinity |
| --- | --- |
| β-ACTIN Monoclonal antibody | 66009, Proteintech |
| Acetyl-Histone H3-K9 Rabbit pAb | A7255, ABclonal |
| Acetyl-Histone H3-K14 Rabbit pAb | A7254, ABclonal |
| Acetyl-Histone H3-K18 Rabbit pAb | A7257, ABclonal |
| Acetyl-Histone H3-K27 Rabbit pAb | A7253, ABclonal |
| human Histone-H3 Polyclonal antibody | 17168, Proteintech |
| KAT2A/GCN5 Monoclonal antibody | 66575-1-Ig, Proteintech |
| KAT2B/PCAF Rabbit mAb | A22719, ABclonal |
| PKNOX2 Polyclonal antibody | 20352, Proteintech |
| IRF4 Monoclonal antibody | 66451, Proteintech |
| Oct6-Specific Polyclonal antibody | 18997, Proteintech |
| CEBPB Monoclonal antibody | 66649, Proteintech |
| MEOX1 Polyclonal antibody | 18190, Proteintech |
| CCL5/RANTES Rabbit pAb | A14192, ABclonal |
| HRP-conjugated Affinipure Goat Anti-Rabbit IgG(H+L) | SA00001-2, Proteintech |
| HRP-conjugated Affinipure Goat Anti-Mouse IgG(H+L) | SA00001-1, Proteintech |
